# Fast and Green Microwave-Assisted Conversion of Essential Oil Allylbenzenes into the Corresponding Aldehydes via Alkene Isomerization and Subsequent Potassium Permanganate Promoted Oxidative Alkene Group Cleavage

**DOI:** 10.3390/molecules14093411

**Published:** 2009-09-03

**Authors:** Thi Xuan Thi Luu, Trinh To Lam, Thach Ngoc Le, Fritz Duus

**Affiliations:** 1 Department of Science, Systems, and Models, Roskilde University, P.O. Box 260, DK-4000, Roskilde, Denmark; E-mail: luu@ruc.dk (T.X.T.L.); 2 Department of Organic Chemistry, University of Science, National University of Ho Chi Minh City, 227 Nguyen Van Cu, Ho Chi Minh City, Vietnam; E-Mail: trinh.lam@bluescopesteel.com (T.T.L.); lenthach@hcm.vnn.vn (T.N.L.)

**Keywords:** microwave irradiation, solvent-free reaction, alkene isomerization, alkene oxidation

## Abstract

Essential oil allylbenzenes from have been converted quickly and efficiently into the corresponding benzaldehydes in good yields by a two-step “green” reaction pathway based on a solventless alkene group isomerization by KF/Al_2_O_3_ to form the corresponding 1-arylpropene and a subsequent solventless oxidation of the latter to the corresponding benzaldehyde by KMnO_4_/CuSO_4_ 5H_2_O. The assistance by microwave irradiation results in very short reaction times (<15 minutes). The green conversion of eugenol (4-allyl-2-methoxyphenol) into vanillin (4-hydroxy-3-methoxybenzaldehyde) has been carried out in a similar way, requiring however two additional microwave-assisted synthetic steps for acetylation of the hydroxy group prior to the oxidation reaction, and for the final deacetylation of vanillin acetate (4-acetoxy-3-methoxybenzaldehyde) by KF/Al_2_O_3_ under solvent-free conditions, respectively.

## Introduction

Allylbenzenes such as *e*.*g*.*,* methylchavicol, safrole, and eugenol are the main ingredients of several essential oils found in plants in tropical areas [1]. The usefulness of these compounds as starting materials in the synthesis of important flavourings has been amply demonstrated. Thus, in 1924 Aoyama and Otake reported the preparation of vanillin from eugenol via KOH promoted alkene group isomerization and subsequent oxidation of isoeugenol by ozonolysis [2]. In 1949 Mayer prepared vanillin by alkene isomerization of eugenol followed by oxidation of isoeugenol by nitrobenzene [3]. In 1992 Bao treated sassafras oil, containing 90% of safrole, with aqueous KOH and oxidized the isomerized product by sodium dichromate in 32% aqueous H_2_SO_4_ [4]. In fact, the synthetic transformation of naturally occurring allylbenzenes into flavouring aromatic aldehydes by alkene group isomerization and subsequent oxidative cleavage of the new C=C double bond has been studied quite extensively, both with respect to the nature of the base catalyst used for alkene isomerization [5,6,7,8,9,10,11,12,13,14,15], and the nature of the oxidation agent used for formation of the aldehyde [15,16,17,18,19,20].

**Scheme 1:**
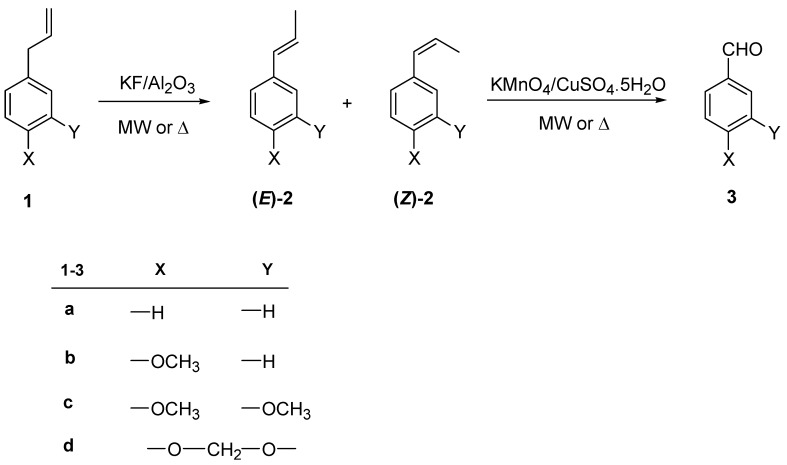
Conversion of allylbenzenes into the corresponding benzaldehydes under solvent-free conditions.

**Scheme 2:**
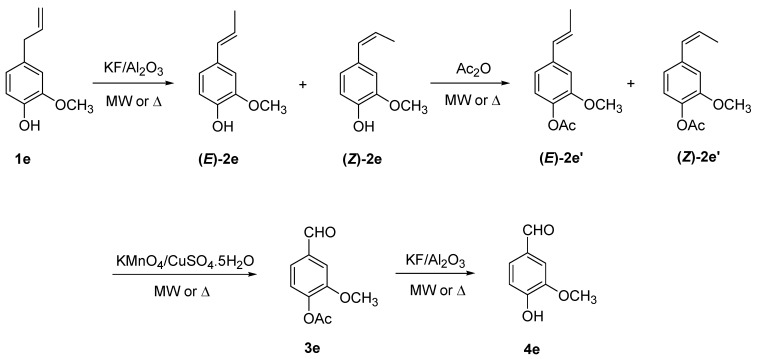
Conversion of eugenol into vanillin under solvent-free conditions.

In recent years the principles of “green chemistry” [21,22,23,24,25,26] have gained increasing attention for the benefit of environmental mildness and savings of materials and energy resources. New concepts such as "green reagents", "solvent-free reactions", and "microwave or ultrasound supported reactions" are now generally accepted and widely used. In this paper we present a new completely green microwave-assisted synthetic procedure for the conversion of allylbenzenes (whether or not procured from essential oils) into the corresponding benzaldehydes in two steps: solvent-free isomerization of the former into the corresponding 1-arylpropenes by KF/Al_2_O_3_, and green solvent-free oxidation of the latter into the benzaldehydes by KMnO_4_/CuSO_4_ 5H_2_O (Scheme 1, Scheme 2). For the conversion of eugenol into vanillin (Scheme 2), two additional subordinate steps were required for protection of the phenolic OH group: microwave-assisted acetylation of isoeugenol by acetic anhydride, and microwave-assisted solvent-free ester hydrolysis [27] by KF/Al_2_O_3_. For comparison, all the above mentioned reactions were also performed under conventional heating conditions. 

## Results and Discussion

### Isomerization of allylbenzenes

In 1990 Radhakrishna and coworkers introduced KF/Al_2_O_3_ as an efficient and versatile reagent for isomerization of olefins under heterogeneous reaction conditions [7]. Three years later, on the basis of a comparative study of the efficiencies of KF/Al_2_O_3_, KOH, and *t*-BuOK as isomerization reagents towards olefins, Le and coworkers called attention to the usefulness of the first mentioned reagent, in particular under solvent-free reaction conditions [8]. The assistance of microwave irradiation was introduced in 1993 by Loupy and Le in connection with their investigation of the base-catalysed isomerization of eugenol [9]. The microwave-assisted isomerization of safrole was reported by Salmoria and his coworkers in 1997 [10]. The applicability of microwave-activated isomerization of organic compounds adsorbed onto inorganic solids was demonstrated by Villemin and coworkers in 1989 [28].

**Figure 1:**
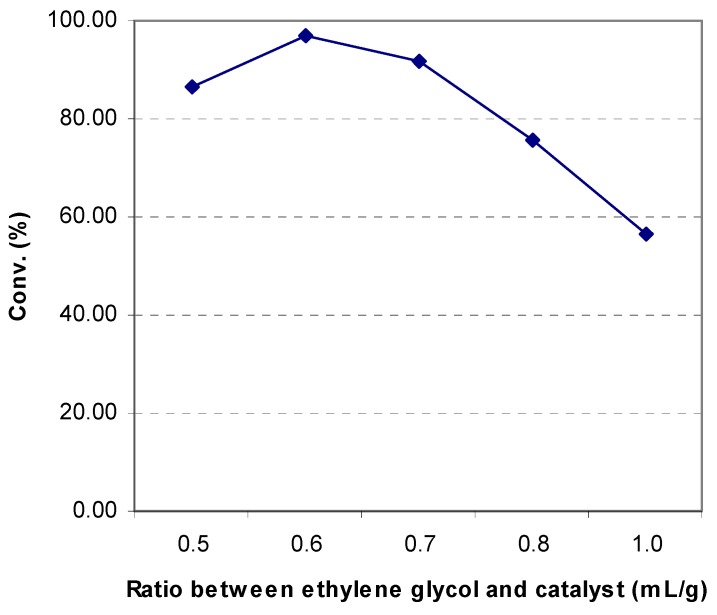
Influence of solvent volume relative to the quantity of KF/Al_2_O_3_ on the efficiency of the isomerization of eugenol into isoeugenol under heterogeneous reaction conditions (eugenol: 2 mmol, KF/Al_2_O_3_: 40 mmol, 5.8 g).

Altogether four allylbenzenes, **1a**-**1d**, were subjected to isomerization by KF/Al_2_O_3_ under solvent-free reaction conditions. Two different methods were used. In Method A (Table 1) the mixture of the allylbenzene and KF/Al_2_O_3_ was irradiated by microwaves for 6-15 minutes. For each of the allylbenzenes, the optimum molar ratio of the allylbenzene and KF/Al_2_O_3_ was determined. In general, high yields were obtained within short reaction times. In method B (Table 1) the same reaction was performed without microwave irradiation, the reaction vessel being instead placed in an oil bath kept at the same temperature as measured for the reaction mixture at the end of the microwave-assisted reaction. The results of Method B (Table 1) clearly demonstrate that high reaction temperatures and long reaction times are required for the complete conversion of allylbenzenes into the isomeric 1-aryl-propenes. On the other hand, the high capacity of KF/Al_2_O_3_ for microwave energy absorption may lead to incipient decomposition of the product before the isomerization is fully completed. The attempted isomerization of eugenol (**1e**) into isoeugenol under solvent-free reaction conditions gave only a low yield of the product (14%), probably due to the extensive deprotonation of the phenolic OH group. However, according to Radhakrishna and his coworkers [7], eugenol can be converted into isoeugenol in the yield of 78% by KF/Al_2_O_3_ upon reflux in ethylene glycol for 90 minutes. Evidently, the solvent ethylene glycol has the effect of being an inhibitor towards phenolic OH group deprotonation. Therefore, the microwave-assisted KF/Al_2_O_3_-promoted isomerization of eugenol into isoeugenol was performed under heterogeneous conditions, using ethylene glycol as the solvent. In order to determine the optimum reaction conditions, a series of experiments were carried out, in which the amount of the solvent was varied in proportion to the amount of KF/Al_2_O_3_ (Figure 1).

All experiments were carefully monitored by GC/MS. Thus, it appeared that the isomerization reactions in all cases gave a mixture of the geometrically isomeric *cis*- and *trans*-1-arylpropenes, **(*Z*)-2** and **(*E*)-2**, respectively. The *trans*-1-arylpropenes were the predominant species in all cases (Table 1). The identity of all products was confirmed by ^1^H- and ^13^C-NMR spectroscopy (Experimental Section).

**Table 1:** Optimized results of allylbenzene isomerization by KF/Al_2_O_3_.

1	1: (KF/Al_2_O_3_) (mol/mol)	Method A^a^			Method B^a^		
		Yield^b ^(Conv.,^b^ time,^c ^P^d^)	Selectivity^b^ (%)		Yield^b^ (Conv.,^b^ time,^c^^∗^ temp^f^)	Selectivity^b^ (%)	
			*cis*	*trans*		*cis*	*trans*
**1a**	2 : 4	96(100, 11, 630)	15	85	99 (100, 2.5, 140)	10	90
**1b**	2 : 10	94 (100, 13, 400)	14	86	96 (100, 3.5, 150)	13	87
**1c**	2 : 4	96 (99, 15, 400)	16	83	99 (100, 2.5, 140)	13	87
**1d**	2 : 8	96 (96, 6, 630)	19	77	98 (100, 2.5, 150)	12	88
**1e**	2 : 40	97 (97, 9, 80)^ a^^∗^	6	91	77 (78, 1.3, 203)^a^^∗∗^	11	67

^a^ Method A: The reaction was assisted by microwave irradiation under solvent-free conditions; Method B: The reaction was assisted by heating in an oil bath under solvent-free conditions; ^a^^∗^ Method A1: The reaction was assisted by microwave irradiation under heterogeneous conditions; ^a^^∗∗^ Method B1: The reaction was assisted by heating in an oil bath under heterogeneous conditions; ^b^ Yield, Conv. (conversion yield), and Selectivity were determined by GC/MS; ^c^ time = reaction time in minutes; ^c^^∗^ time = reaction time in hours; ^d^ P = power of microwave oven (W); ^f^ temp = oil bath temperature (°C).

### Oxidation of 1-arylpropenes

In 1979 Menger and Lee announced that under heterogeneous conditions a mixture of KMnO_4_ and CuSO_4_ 5H_2_O was unreactive towards the carbon-carbon double bond in the oxidation reaction of unsaturated secondary alcohols into the corresponding ketones using the above mentioned reagent [29]. Nevertheless, inspired by later reports on successful KMnO_4_ promoted oxidation reactions of 1-aryl-1-alkenes [17,19], we investigated the possibility of using the mixture of KMnO_4_ and CuSO_4_**∙**5H_2_O for oxidation of 1-phenylpropene (our pertinently chosen model substance) into benzaldehyde under solvent-free reaction conditions. In order to find the most efficient oxidation agent, a series of experiments was performed, where the molar ratio between KMnO_4_ and CuSO_4_ 5H_2_O was varied. The most efficient of the oxidants investigated (all of them being prepared by the vaporization method [30]), appeared to be KMnO_4_ absorbed on a fourfold molar amount of CuSO_4_ 5H_2_O. This oxidant, in the following referred to as **PP/4CSP** (**p**otassium **p**ermanganate absorbed on a **fourfold **molar amount of **c**opper **s**ulfate **p**entahydrate) was chosen as the standard oxidant in our subsequent experiments. In order to achieve the very best yield of benzaldehyde, we paid attention also to the molar ratio between 1-phenylpropene and **PP/4CSP**. The most appropriate molar ratio (3:12) was also chosen for the oxidation of the four other 1-arylbenzenes. All 1-arylpropenes used were mixtures of the geometrically isomeric forms (Table 1).

The solvent-free oxidation of the 1-arylpropenes into the corresponding benzaldehydes was performed under the assistance of microwave irradiation (Method C), as well as under conventional heating conditions (Method D). The results are listed in Table 2. For both methods, it is clear that the 1-arylpropenes containing electron donating substituents are oxidized most efficiently. Not surprisingly, the microwave-assisted oxidations proceed markedly faster than the corresponding reactions driven by conventional heating. However, the yields obtained by Method C also appear to be generally higher than those of Method D. Minor amounts of overoxidation products were easily removed by filtration of the crude product mixture through a layer of NaHCO_3_/SiO_2_ (Experimental Section). Thus the 1-arylpropenes **2a**-**2d** could be smoothly converted into the aldehydes **3a**-**3d**.

However, upon treatment with **PP**/**4CSP**, isoeugenol (**2e)** partly decomposed with the development of some black smoke. Obviously, the phenolic OH group of isoeugenol should be protected. Thus a mixture of isoeugenol and a minor excess of acetic anhydride was reacted to form isoeugenol acetate (**2e’**, Scheme 2) under the assistance of microwave irradiation, as well as under conventional heating conditions. As expected, the crucial factor for obtaining maximum yield by both methods is the reaction time, which was found to be considerably shorter for the microwave-assisted reaction. The 100% conversion of isoeugenol (**2e**) into isoeugenol acetate (**2e’**) under the assistance of microwave irradiation (80 W) could be performed within 20 minutes, whereas the same reaction supported by conventional heating at 150 °C required 90 minutes. 

**Table 2:** Oxidation of 1-arylpropenes into corresponding benzaldehydes.

2 (mmol)	KMnO_4_ (mmol)	CuSO_4_ 5H_2_O (mmol)	Method C^a^	Method D^a^
			Yield^b^ (Conv.,^b^ time,^c^ P^d^)	Yield^b^ (Conv.,^b^ time,^c^ temp^f^)
**2a** (1.5)	6	24	52 (60, 8, 240)	50 (57, 60, 90)
**2b** (1.5)	6	24	70 (86, 7.3, 350)	80 (95, 60, 96)
**2c** (1.5)	6	24	79 (96, 7.5, 350)	75 (94, 13, 100)
**2d** (1.5)	6	24	73 (90, 17, 450)	77 (93, 60, 95)
**2e’**(1.5)	6	24	58 (77, 16, 450)	56 (75, 90, 94)

^a^ Method C: The reaction was assisted by microwave irradiation; Method D: The reaction was assisted by heating in an oil bath at the appropriate temperature; ^b^ Yield and Conv. (conversion yield) were determined by GC/MS; ^c^ time = reaction time in minutes; ^d^ P = power of microwave oven (W); ^f^ temp = oil bath temperature (°C)

The oxidation of isoeugenol acetate (**2e’**) into vanillin acetate (**3e**; Scheme 2) was performed in the same way as for the other 1-arylpropenes **2a**-**2d **(Table 2), *i*.*e*.*,* under the assistance of microwave irradiation (Method C), as well as with provision of energy from conventional heating (Method D). The yields of **3e** were in both cases higher than the corresponding yields of benzaldehyde (**3a**), but still lower than the corresponding yields of the aldehydes **2b**-**2d**. Evidently, the presence of the acetoxy group has a hindering influence on the oxidation sensitivity of **2e’**.

**Figure 2:**
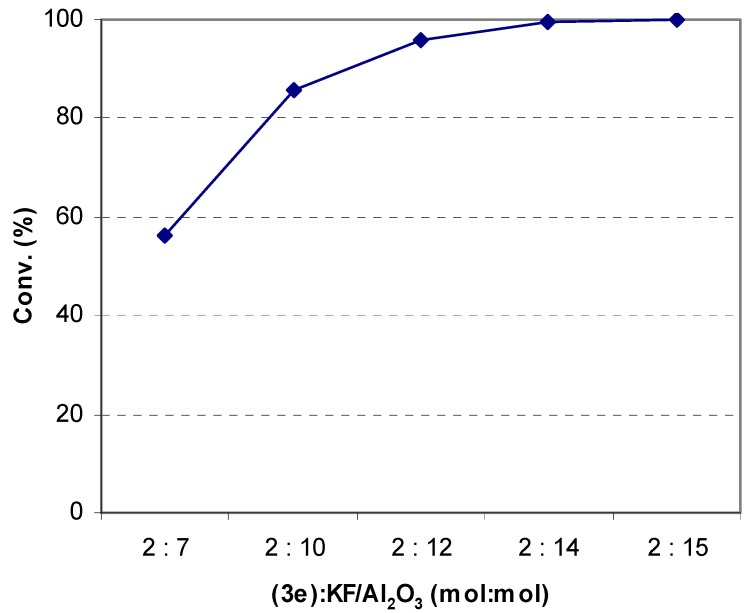
Influence of the molar ratio between vanillin acetate and KF/Al_2_O_3_ on the efficiency of the microwave-assisted hydrolysis of vanillin acetate into vanillin under solvent-free reaction conditions (irradiation time: 14.5 minutes; power: 300 W).

The last step, the hydrolysis of vanillin acetate (**3e**) into vanillin (**4e**), was carried out under solvent-free conditions using KF/Al_2_O_3_ as base catalyst [27]. In order to determine the reaction conditions leading to the highest yield, the influence of the molar ratio between vanillin acetate and KF/Al_2_O_3_ was investigated initially under intuitively chosen microwave irradiation conditions (Figure 2). Subsequently, the optimum conditions with respect to the duration of the microwave irradiation were determined (Table 3, Method E). For comparison, a series of hydrolysis experiments was carried out under conditions of conventional heating (Table 3, Method F). Thus, while the 100% conversion of vanillin acetate into vanillin with the assistance of microwave irradiation could be performed within 14 minutes, the same reaction supported by conventional heating required about one hour (Table 3).

**Table 3:** Optimization of the hydrolysis of vanillin acetate by KF/Al_2_O_3_.

Method^a^	Time (min)	Temp. (°C)^c^	Conv.^b^ (%)	Yield^b ^(%)
Method E	14.5	110	100	92
Method E	14.0	115	100	94
Method E	13.0	122	99.5	96
Method F	14	115	82	76
Method F	30	115	97	91
Method F	60	115	100	91

^a^ Method E: The hydrolysis was assisted by microwave irradiation at 300 W; Method F: The hydrolysis was supported by conventional heating to 115 °C; ^b^ Conv. and yield were determined by GC/MS; ^c^ Temp. = temperature measured when the microwave irradiation was stopped (Method E); Temp. = oil bath temperature (Method F)

The identity and purity of all products reported were confirmed by ^1^H-NMR and ^13^C-NMR spectroscopy, as well as by GC/MS. Since all benzaldehyde products are well-known and already spectroscopically well-characterized [31,32,33,34,35], a further presentation and discussion of their spectral characteristics has been found superfluous. On the other hand, the 1-arylpropenes formed (as mixtures of geometrically isomeric *cis*-and *trans*-forms) by the isomerization of the allylbenzenes appear not to have been well-characterized spectroscopically [36,37], for which reason full details of both the ^1^H- NMR spectra (600 MHz) and the ^13^C-NMR spectra are presented in the Experimental.

## Experimental

### General

Microwave irradiations were performed by means of a CEM MDS 200 batch microwave oven. GC/MS analyses were performed on a Hewlett Packard 5890 GC 5971A MS apparatus equipped with a J&W DB-5MS capillary column (30 m, 0.25 mm i.d., 0.25 μm film thickness) and a Hewlett Packard 7673A autosampler. NMR spectra were recorded on a Varian Mercury 300 NMR spectrometer and/or a Varian Inova 600 NMR spectrometer on ca. 0.2 M solutions (solvent: CDCl_3_) at 25 °C. All chemicals used were from obtained from Aldrich or Fluka.

### Preparation of base catalyst KF/Al_2_O_3_ (40% w/w)

KF (20 g) was dissolved completely in de-ionized water (150 mL, pH of solution: 6.5). Al_2_O_3_ (30 g) was stirred regularly in de-ionized water (150 mL) for around 5 minutes (pH of solution: 7.6). Then the KF solution was poured into the solution containing Al_2_O_3_ under continuous stirring for 30-45 minutes, until the pH of the mixture of the solutions was 11.5-11.7. Subsequently, water was removed from the solution mixture by rotatory evaporation, until the weight of the remaining solid mass was 53-55 g. The wet solid mass was dried under vacuum (200 mbar) in an oven at 140-150 °C for 6 hours. Finally, the obtained solid mass (50.5-51.5 g) was ground in a mortar into a fine homogeneous powder (*Note*: the use of laboratory protective gloves and an inhalation mask is recommended!). 

### Preparation of oxidation agent PP/4CSP

Copper sulfate pentahydrate (0.1 mol, 25 g) was dissolved completely in de-ionized water. Then KMnO_4_ (0.025 mol, 3.95 g) was added, followed by a sufficient volume of de-ionized water to obtain a homogeneous solution. The solution was stirred for 10 minutes at 80 °C. Subsequently water was removed from the solution by rotatory evaporation, until the weight of the remaining solid mass was equal to the sum of the weights of the original ingredients. The obtained solid mass was ground in a mortar into a fine homogeneous powder (*Note*: the use of laboratory protective gloves and an inhalation mask is recommended!).

### Isomerization of allylbenzenes into the corresponding 1-arylpropenes by KF/Al_2_O_3_ under solvent-free reaction conditions with microwave irradiation (Method A)

A suitable quantity of finely ground KF/Al_2_O_3_ was added to a test tube (h = 16 cm, d = 1.4 cm; alternatively: h = 20 cm, d = 3 cm) containing the allylbenzene (2.00 mmol). The test tube was placed in an alumina bath, equipped to support test tubes, in the microwave oven. For each of the allylbenzenes, an irradiation programme was applied to determine the most efficient reaction conditions, see Table 1. For every experiment performed, the temperature of the reaction mixture was measured immediately after the termination of the reaction. After cooling, the reaction mixture was extracted with ether (4 × 15 mL). The combined extracts were filtered, washed with water until neutral, and then dried by anhydrous Na_2_SO_4_. After removal of the solvent by rotatory evaporation, the remaining crude product was analysed by GC/MS and NMR spectroscopy.

### Isomerization of allylbenzenes into the corresponding 1-arylpropenes by KF/Al_2_O_3_ under solvent-free reaction conditions with conventional heating (Method B)

A suitable quantity of finely ground KF/Al_2_O_3_ was added to a test tube (h = 16 cm, d = 1.4 cm; alternatively: h = 20 cm, d = 3 cm) containing the allylbenzene (2.00 mmol). The test tube was placed in an oil bath heated to the desired temperature, and kept there for the pertinent period of time, see Table 1. Subsequently, the reaction mixture was worked up as described in Method A.

### Isomerization of eugenol into isoeugenol by KF/Al_2_O_3_ under heterogeneous reaction conditions with microwave irradiation (Method A1)

The necessary volume of ethylene glycol [calculated on the basis of the quantity (g) of KF/Al_2_O_3_] was added into a test tube (h = 20 cm, d = 3 cm) containing the finely ground KF/Al_2_O_3_, and then eugenol (0.328 g, 2.00 mmol) was added under shaking. The test tube was placed into a beaker equipped to support test tubes and placed in the microwave oven. An irradiation programme was applied to determine the most efficient reaction conditions, see Table 1. For every experiment performed, the temperature of the reaction mixture was measured immediately after the end of the reaction. After cooling, the reaction mixture was dissolved in water (50 mL), and the aqueous solution was neutralized (pH = 7) with 10% aqueous HCl. Then toluene (50 mL) was added, and the new solution mixture was stirred for 15-30 minutes, and then filtered through a glass filter funnel. The two-layer filtrate was separated by means of a separating funnel. The toluene layer was washed with water, and then dried by anhydrous Na_2_SO_4_. After removal of the solvent by rotational evaporation, the remaining crude product was analysed by GC/MS and NMR spectroscopy. The pure isoeugenol (as a mixture of *cis* and *trans* isomeric forms) was isolated by flash column chromatography (4-7 g silica gel, Davisil, grade 710, 4-20 μm, 60 A, 99%) using as eluent a mixture of hexane and dichloromethane (9:1 v/v).

### Isomerization of eugenol into isoeugenol by KF/Al_2_O_3_ under heterogeneous reaction conditions with conventional heating (Method B1)

The pertinent volume of ethylene glycol was poured into a test tube (h = 20 cm, d = 3 cm) containing the finely ground KF/Al_2_O_3_, and then eugenol (0.328 g, 2.00 mmol) was added under shaking. The test tube was placed in an oil bath heated to the temperature measured by the reaction stop of the parallel reaction run under microwave irradiation, and kept there for the pertinent period of time. After cooling, the reaction mixture was worked up as described in Method A1.

### Oxidation of 1-arylpropenes into the corresponding aldehydes by PP/4CSP under solvent-free reaction conditions with microwave irradiation (Method C)

Finely ground **PP/4CSP** (13.896 g, 12.00 mmol) was added to a test tube (h = 20 cm, d = 3.0 cm) containing the 1-arylpropene (3.00 mmol). The test tube was placed into a beaker, equipped to support test tubes in the microwave oven. For each of the 1-arylpropenes, an irradiation programme was applied to determine the most efficient reaction conditions, see Table 2. For every experiment performed, the temperature of the reaction mixture was measured immediately after the irradiation ceased. After cooling, the reaction mixture was stirred with diethyl ether (50 mL) for 15 minutes, and then filtered through a layer of Celite (2 cm). The ethereal extract was concentrated by rotational evaporation until a total volume of around 10 mL, and then filtered through a layer of NaHCO_3_/SiO_2 _ (3 cm). The layer of NaHCO_3_/SiO_2_ was rinsed with diethyl ether (10-15 mL), after which the combined ethereal layers were dried by anhydrous Na_2_SO_4_. After removal of the solvent by rotatory evaporation, the remaining crude product was analysed by GC/MS and NMR spectroscopy. The aldehyde was isolated by flash column chromatography (4-7 g silica gel, Davisil, grade 710, 4-20 μm, 60 A, 99%) using as eluent a mixture of hexane and ethyl acetate (9:1 v/v).

### Oxidation of 1-arylpropenes into the corresponding aldehydes by PP/4CSP under solvent-free reaction conditions with conventional heating (Method D)

A test tube (h = 20 cm, d = 3.0 cm) containing finely ground **PP/4CSP** (13.896 g, 12.00 mmol) and the 1-arylpropene (3.00 mmol) was placed in an oil bath heated to the temperature measured by reaction stop of the parallel reaction run under microwave irradiation, and kept there for the pertinent period of time. After cooling, the reaction mixture was worked up as described in Method C. 

### Hydrolysis of vanillin acetate into vanillin by KF/Al_2_O_3_ under solvent-free reaction conditions with microwave irradiation (Method E)

A suitable quantity of finely ground KF/Al_2_O_3_ was added to a test tube (h = 16 cm, d = 1.4 cm) containing vanillin acetate (2.00 mmol). The test tube was placed into a beaker, equipped to support test tubes in the microwave oven. An irradiation programme was applied to determine the most efficient reaction conditions. For every experiment performed, the temperature of the reaction mixture was measured immediately after the irradiation was stopped. After cooling, the reaction mixture was dissolved in water (50 mL) and the aqueous solution was neutralized with 10% aqueous HCl. Then dichloromethane (50 mL) was added, the two-layer solution mixture was stirred for 15-30 minutes, and filtered through a glass filter funnel. The dichloromethane layer was separated and dried by anhydrous Na_2_SO_4_. After removal of the solvent by rotational evaporation, the remaining crude product was analysed by GC/MS and NMR spectroscopy. Vanillin was isolated by flash column chromatography (4-7 g silica gel, Davisil, grade 710, 4-20 μm, 60 A, 99%) using as eluent a mixture of dichloromethane and ethyl acetate (9:1 v/v).

### Hydrolysis of vanillin acetate into vanillin by KF/Al_2_O_3_ under solvent-free reaction conditions with conventional heating (Method F)

A suitable quantity of finely ground KF/Al_2_O_3_ was added into a test tube (h = 16 cm, d = 1.4 cm) containing vanillin acetate (2.00 mmol). The test tube was placed in an oil bath heated to the temperature measured by reaction stop of the parallel reaction run under microwave irradiation, and kept there for the pertinent period of time. After cooling, the reaction mixture was worked up as described in Method E.

### ^1^H- and ^13^C-NMR spectroscopic data

*(E)-1-phenylpropene* **(*E*)-2a**: ^1^H-NMR (600 MHz) δ (ppm) = 7.31 (dd, *J* = 8.4 Hz, *J* = 1.3 Hz, 2H), 7.26 (dd, *J* = 8.4 Hz, *J* = 7.2 Hz, 2H), 7.16 (tt, *J* = 7.2 Hz, *J* = 1.3 Hz, 1H), 6.38 (dq, *J* = 15.6 Hz, *J* = 1.2 Hz, 1H), 6.22 (dq, *J* = 15.6 Hz, *J* = 6.6 Hz, 1H), 1.86 (dd, *J* = 6.6 Hz, *J* = 1.2 Hz, 3H); ^13^C-NMR (75 MHz) δ (ppm) = 137.95, 131.05, 128.47, 126.73, 125.82, 125.66, 18.47.

*(Z)-1-phenylpropene* **(*Z*)-2a**: ^1^H-NMR (600 MHz) δ (ppm) = 7.30 (dd, *J* = 8.4 Hz, *J* = 1.5 Hz, 2H), 7.26 (dd, *J* = 8.4 Hz, *J* = 7.2 Hz, 2H), 7.20 (tt, *J* = 7.2 Hz, *J* = 1.5 Hz, 1H), 6.43 (dq, *J* = 11.4 Hz, *J* = 1.8 Hz, 1H), 5.78 (dq, *J* = 11.4 Hz, *J* = 7.2 Hz, 1H), 1.89 (dd, *J* = 7.2 Hz, *J* = 1.8 Hz, 3H); ^13^C-NMR (75 MHz) δ (ppm) = 137.62, 129.87, 128.83, 128.11, 126.76, 126.41, 14.61. 

*(E)-1-(4-Methoxyphenyl)propene* **(*E*)-2b**: ^1^H-NMR (600 MHz) δ (ppm) = 7.24-7.26 (m, 2H), 6.81-6.83 (m, 2H), 6.33 (dq, *J* = 16.0 Hz, *J* = 1.8 Hz, 1H), 6.08 (dq, *J* = 16.0 Hz, *J* = 6.6 Hz, 1H), 3.78 (s, 3H), 1.85 (dd, *J* = 6.6 Hz, *J* = 1.8 Hz, 3H); ^13^C-NMR (75 MHz) δ (ppm) = 158.59, 130.82, 130.35, 126.88, 123.47, 113.90, 55.25, 18.41. 

*(Z)-1-(4-Methoxyphenyl)propene* **(*Z*)-2b**: ^1^H-NMR (600 MHz) δ (ppm) = 7.22-7.24 (m, 2H), 6.86-6.88 (m, 2H), 6.36 (dq, *J* = 11.4 Hz, *J* = 1.8 Hz, 1H), 5.69 (dq, *J* = 11.4 Hz, *J* = 7.2 Hz, 1H), 3.79 (s, 3H), 1.88 (dd, *J* = 7.2 Hz, *J* = 1.8 Hz, 3H); ^13^C-NMR (75 MHz) δ (ppm) = 158.12, 130.34, 129.89, 129.28, 125.09, 113.55, 55.23, 14.59. 

*(E)-1-(3,4-Dimethoxyphenyl)propene* **(*E*)-2c**: ^1^H-NMR (600 MHz) δ (ppm) = 6.89 (d, *J* = 1.8 Hz, 1H), 6.85 (dd, *J* = 8.4 Hz, *J* = 1.8 Hz, 1H), 6.79 (d, *J* = 8.4 Hz, 1H), 6.33 (dq, *J* = 15.6 Hz, *J* = 1.6 Hz, 1H), 6.10 (dq, *J* = 15.6 Hz, *J* = 6.6 Hz, 1H), 3.88 (s, 3H), 3.86 (s, 3H), 1.86 (dd, *J* = 6.6 Hz, *J* = 1.6 Hz, 3H); ^13^C-NMR (75 MHz) δ (ppm) = 148.99, 148.17, 131.16, 130.62, 123.77, 118.66, 111.20, 108.49, 55.91, 55.77, 18.37. 

*(Z)-1-(3,4-Dimethoxyphenyl)propene* **(*Z*)-2c**: ^1^H-NMR (600 MHz) δ (ppm) = 6.87 (d, *J* = 1.8 Hz, 1H), 6.83-6.85 (m, 2H), 6.37 (dq, *J* = 11.4 Hz, *J* = 1.8 Hz, 1H), 5.71 (dq, *J* = 11.4 Hz, *J* = 7.2 Hz, 1H), 3.878 (s, 3H), 3.877 (s, 3H), 1.91 (dd, *J* = 7.2 Hz, *J* = 1.8 Hz, 3H); ^13^C-NMR (75 MHz) δ (ppm) = 148.52, 147.67, 131.16, 129.53, 125.40, 121.36, 112.17, 110.93, 55.88, 55.80, 14.66. 

*(E)*-*5-(Prop-1-enyl)benzo[d]-1,3-dioxole* [*(E)-Isosafrol*, **(*E*)-2d**]: ^1^H-NMR (600 MHz) δ (ppm) = 6.87 (d, *J* = 1.2 Hz, 1H), 6.73 (dd, *J* = 8.1 Hz, *J* = 1.2 Hz, 1H), 6.72 (d, *J* = 8.1 Hz, 1H), 6.30 (dq, *J* = 15.9 Hz, *J* = 1.8 Hz, 1H), 6.05 (dq, *J* = 15.9 Hz, *J* = 6.6 Hz, 1H), 5.91 (s, 2H), 1.84 (dd, *J* = 6.6 Hz, *J* = 1.8 Hz, 3H); ^13^C-NMR (75 MHz) δ (ppm) = 147.92, 146.51, 132.51, 130.57, 123.92, 120.06, 108.20, 105.34, 100.90, 18.33. 

*(Z)-5-(Prop-1-enyl)benzo[d]-1,3-dioxole* [*(Z)-Isosafrol*, **(*Z*)-2d**]: ^1^H-NMR (600 MHz) δ (ppm) = 6.82 (d, *J* = 1.2 Hz, 1H), 6.78 (d, *J* = 7.8 Hz, 1H), 6.75 (dd, *J* = 7.8 Hz, *J* = 1.2 Hz, 1H), 6.35 (dq, *J* = 11.4 Hz, *J* = 1.8 Hz, 1H), 5.94 (s, 2H), 5.69 (dq, *J* = 11.4 Hz, *J* = 7.2 Hz, 1H), 1.87 (dd, *J* = 7.2 Hz, *J* = 1.8 Hz, 3H); ^13^C-NMR (75 MHz) δ (ppm) = 147.40, 146.01, 131.79, 129.45, 125.59, 122.53, 109.05, 108.08, 100.90, 14.61.

*(E)-1-(4-Hydroxy-3-methoxyphenyl)propene*, [*(E)-Isoeugenol*, **(*E*)-2e**]: ^1^H-NMR (600 MHz) δ (ppm) = 6.80-6.86 (m, 3H), 6.31 (dq, *J* = 15.6 Hz, *J* = 1.8 Hz, 1H), 6.07 (dq, *J* = 15.6 Hz, *J* = 6.6 Hz, 1H), 5.59 (s, 1H), 3.87 (s, 3H), 1.84 (dd, *J* = 6.6 Hz, *J* = 1.8 Hz, 3H); ^13^C-NMR (75 MHz) δ (ppm) = 146.57, 144.76, 130.75, 130.66, 123.42, 119.31, 114.37, 107.90, 55.83, 18.34.

*(Z)-1-(4-Hydroxy-3-methoxyphenyl)propene* [*(Z)-Isoeugenol*, **(*Z*)-2e**]: ^1^H-NMR (600 MHz) δ (ppm) = 6.89 (d, *J* = 8.4 Hz, 1H), 6.83-6.86 (m, 1H), 6.80 (d, *J* = 1.8 Hz, 1H), 6.35 (dq, *J* = 15.6 Hz, *J* = 1.8 Hz, 1H), 5.69 (dq, *J* = 12.0 Hz, *J* = 7.2 Hz, 1H), 5.63 (s, 1H), 3.86 (s, 3H), 1.89 (dd, *J* = 7.2 Hz, *J* = 1.8 Hz, 3H); ^13^C-NMR (75 MHz) δ (ppm) = 146.14, 144.29, 130.08, 129.64, 125.14, 122.09, 114.10, 111.43, 55.87, 14.64.

*(E)*-*1-(4-Acetoxy-3-methoxyphenyl)propene*, [*(E)-Isoeugenol acetate*, ***(E)*** **-2e’**]: ^1^H-NMR (600 MHz) δ (ppm) = 6.94 (d, *J* = 8.2 Hz, 1H), 6.92 (d, *J* = 1.8 Hz, 1H), 6.88 (dd, *J* = 8.2 Hz, *J* = 1.8 Hz, 1H), 6.36 (dq, *J* = 15.6 Hz, *J* = 1.2 Hz, 1H), 6.18 (dq, *J* = 15.6 Hz, *J* = 6.6 Hz, 1H), 3.82 (s, 3H), 2.29 (s, 3H), 1.87 (dd, *J* = 6.6 Hz, *J* = 1.2 Hz, 3H); ^13^C-NMR (75 MHz) δ (ppm) = 169.13, 151.00, 138.60, 137.07, 130.46, 126.06, 122.66, 118.36, 109.63, 55.76, 20.64, 18.40.

*(Z)*-*1-(4-Acetoxy-3-methoxyphenyl)propene*, [*(Z)-Isoeugenol acetate*, ***(Z)*** **-2e’**]: ^1^H-NMR (600 MHz) δ (ppm) = 6.99 (d, *J* = 8.4 Hz, 1H), 6.87-6.94 (m, 2H), 6.39 (dq, *J* = 12.0 Hz, *J* = 1.8 Hz, 1H), 5.78 (dq, *J* = 12.0 Hz, *J* = 7.2 Hz, 1H), 3.82 (s, 3H), 2.31 (s, 3H), 1.90 (dd, *J* = 7.2 Hz, *J* = 1.8 Hz, 3H); ^13^C-NMR (75 MHz) δ (ppm) = 166.40, 150.65, 138.19, 136.57, 129.33, 127.06, 122.35, 121.25, 112.97, 55.80, 22.12, 14.62.
